# Exploring the Restorative Effects of Natural Environments in Virtual Reality

**DOI:** 10.3390/ijerph22040535

**Published:** 2025-04-01

**Authors:** Silvia Marocco, Valeria Vitale, Elena Grossi, Fabio Presaghi, Marino Bonaiuto, Alessandra Talamo

**Affiliations:** 1 Department of Social and Developmental Psychology, Sapienza University of Rome, 00185 Rome, Italy; elena.grossi@uniroma1.it (E.G.); fabio.presaghi@uniroma1.it (F.P.); marino.bonaiuto@uniroma1.it (M.B.); alessandra.talamo@uniroma1.it (A.T.); 2 Department of Cognition, Emotion, and Methods in Psychology, University of Vienna, 1010 Vienna, Austria; valeria.vitale@univie.ac.at; 3 CIRPA—Centre for Interuniversity Research in Environmental Psychology, 00185 Rome, Italy

**Keywords:** nature exposure, virtual reality, emotions, valence, arousal

## Abstract

Natural environments are known to foster positive emotions and well-being, while Virtual Reality (VR) represents a powerful tool to explore such effects. This study investigates the restorative potential of virtual natural environments for emotional recovery, combining self-report and physiological measures. Fifty-two participants were divided into three Natural groups—formed spontaneously based on their preferred natural scenarios—and a Neutral group—exposed to a neutral scenario. The results reveal that virtual natural scenarios effectively restored positive emotions (valence) after exposure to arousing stimuli. Both neutral and natural scenarios reduced arousal, but the physiological data show higher activation in Natural groups. Interestingly, this activation was positively appraised, supporting emotional recovery. These findings confirm the restorative potential of VR nature, with nuanced arousal effects.

## 1. Introduction

### 1.1. Benefits of Virtual Natural Environments

The literature in the field of Environmental Psychology, defined as the discipline that deals with interactions and relationships between people and their environment [1], has increasingly focused on the environmental influence on individual and societal health and well-being. In particular, a specific research trend has concentrated on the restorative properties of natural environments, which are widely recognized for their benefits. Engaging with nature provides advantages across multiple health dimensions: physiological, physical, cognitive, social, and emotional. Physiologically, nature helps regulate heart rate (HR) and blood pressure, reduce muscle tension, and lower cortisol levels [2,3]. Emotionally, nature tends to alleviate stress, depression, anxiety, and negative emotions while promoting positive affect and overall well-being [4,5,6,7], with effects persisting over time [8,9]. In particular, experimental research has demonstrated that exposure to natural environments directly influences emotional processes by reducing negative emotions and enhancing positive ones [10,11,12,13]. Additionally, nature indirectly supports emotional regulation by restoring attention, thereby improving executive functioning and self-regulation [10,14,15]. This restoration fosters constructive reflection on unresolved issues [16,17]. Thus, natural stimuli effectively impact both emotional states [6] and emotion regulation [18,19,20].

Several theories explain the restorative power of nature. The Stress Reduction Theory (SRT) posits that humans have an evolutionary preference for natural environments, which reduces stress by lowering physiological arousal and negative emotions [3]. The Attention Restoration Theory (ART) suggests that nature supports mental health by restoring attention through effortless engagement with natural stimuli, offering a break from cognitive demands [14,17].

Although real-world natural environments provide greater well-being benefits compared to indirect experiences [21], much of the evidence supporting nature’s therapeutic potential comes from studies using digital representations [6]. For example, nature videos have proven effective in aiding recovery from physiological and psychological stress markers over the past 30 years [22,23]. Even static images of natural scenes can significantly enhance mood, restore attention, and improve executive function [10,15,24,25].

Research into digital nature experiences via images, videos, and projections has shown their regenerative potential [21], although they often lack the immersive qualities of actual natural settings [26]. These limitations include reduced scale, the inability to transport individuals from their current surroundings, and a lack of multi-sensory engagement [27,28].

Advances in Virtual Reality (VR) technology present a solution to some of these limitations, offering immersive simulations of natural environments [29,30,31,32]. The benefits of VR nature experiences have been linked to improved emotional states, restorativeness, stress relief, and creativity [33,34,35,36,37].

Gentile et al. [38], in their systematic review of the stress-reduction effects of virtual reality natural environments, highlighted a high heterogeneity in the stress induction methods used across studies. They categorized these methods into physical and psychological stressors. Physical stressors included sensory stimuli such as electrical shocks, loud noises, horror movies, and recalling stressful episodes. Psychological stressors encompassed cognitive and social challenges, such as arithmetic tasks, the Trier Social Stress Test—inducing anticipatory stress through a social evaluation followed by a mathematical test—and the PASAT, which involves mental calculations with a triggered siren for incorrect responses to heighten stress. However, a notable gap remains—most studies rely on external stress induction before VR exposure, rather than incorporating stress induction directly within the VR environment. This limitation may reduce the ecological validity and immersiveness of the experience, restraining experimental control and realism.

Moreover, several studies have examined individuals’ responses to virtual nature exposure and their ability to recover following activating or stressful stimuli [39,40,41]. However, a less explored aspect in the literature concerns the use of a procedure involving a sequence of stressful stimuli with increasing intensity, rather than the application of a single stimulus. This approach would allow for a more detailed and comparative evaluation of the restorative potential of natural scenarios, taking into account differences in individual responses to progressively higher stress levels. Another limitation of the existing literature relates to the assignment of natural scenarios. In most studies, scenarios are randomly pre-assigned by the experimenter, without considering participants’ personal preferences. Introducing a recipient-designed and user-centered procedure [42,43,44,45] that allows participants to choose the natural scenario based on their preferences could significantly improve the ecological validity of such studies.

Hence, this study seeks to address these gaps by integrating graded arousal protocols and enabling personalized environment selection, advancing research on the restorative effects of preferred virtual natural environments.

### 1.2. Research Objectives

Thus, within the scope of exploring the restorative effect of virtual natural environments in terms of elicited emotions and physiological responses, this study adopts a methodological approach that combines explicit and implicit measures. The primarily addressed research objectives are as follows:

1. The validation of three natural VR scenarios, assessing their effects on elicited emotional states in terms of arousal and valence;
2. The assessment of the effectiveness of using natural VR scenarios in restoring an emotional baseline after exposure to arousing scenarios.

### 1.3. Research Hypotheses

Based on these research goals, the following hypotheses were formulated.

**H1.**  *Differences between the VR natural scenario and VR neutral scenario.*

**H1a.**  *Valence: Given the evidence in the literature of the effects of natural scenarios on emotional states, we expect people to show higher levels of positive emotions when exposed to VR natural scenarios as opposed to people exposed to VR neutral scenarios.*

**H1b.**  *Perceived arousal: Based on the literature on the stress-relief effects of natural scenarios, we expect people exposed to VR natural scenarios to show less arousal emotional states than people exposed to VR neutral scenarios.*

**H1c.**  *Physiological arousal: As suggested by prior studies, we expect people to show lower HR levels and higher heart rate variability (HRV) when exposed to VR natural scenarios compared to people exposed to VR neutral scenarios.*

**H2.**  *Recovery effects of natural scenarios after exposure to arousing stimuli.*

**H2a.**  *Changes in valence: We expect people who have experienced arousing stimuli to show higher perceived positive emotions when successively exposed to natural scenarios as opposed to people exposed to neutral scenarios.*

**H2b.**  *Changes in perceived arousal: We expect people exposed to arousing stimuli to show lower perceived arousal levels when exposed successively to VR natural scenarios as opposed to people exposed to neutral scenarios.*

**H2c.**  *Changes in physiological arousal: We expect people exposed to an arousing situation to show lower HR levels and higher HRV when successively exposed to VR natural scenarios as opposed to people exposed to neutral scenarios.*

**H3.**  *Effectiveness of natural scenarios in recovering baseline values.*

**H3a.**  *Effectiveness in terms of valence: We hypothesize that in participants’ exposed to natural scenarios (as opposed to the neutral scenario) during the recovery phase, the emotional valence will be restored after the arousing stimuli cycle, returning to the baseline level.*

**H3b.**  *Effectiveness in terms of perceived arousal: We hypothesize that in participants’ exposed to natural scenarios (as opposed to the neutral scenario) during the recovery phase, the emotional arousal will be restored after the arousing stimuli cycle, returning to the baseline level.*

**H3c.**  *Effectiveness in terms of physiological arousal: We hypothesize that in participants’ exposed to natural scenarios (as opposed to the neutral scenario) during the recovery phase, the physiological arousal will be restored after the arousing stimuli cycle, returning to the baseline level.*

To test these hypotheses, an experimental study was conducted with three experimental groups (“Natural groups”), each exposed to their preferred virtual natural environments and compared to a control group (“Neutral group”) exposed to a neutral scenario (a grey screen in VR). A grey screen in VR was selected as the “neutral” scenario due to the grey color’s un-emotional connotations [46]. The natural and neutral scenarios are referred to as “recovery scenarios”, as they were introduced following arousing stimuli. In this study, virtual war-related scenarios were selected as the arousing stimuli, overcoming the existing literature gap previously presented.

## 2. Materials and Method

### 2.1. Research Design

This study employed a mixed research design consisting of four distinct phases and four independent groups of participants, as follows: a pre-test measurement phase for physiological data, aimed at ensuring no differences exist in ECG activity among participants assigned to the different groups; a baseline phase for the natural scenarios, intended to assess participants’ emotional and physiological reactions to the proposed natural scenarios; a reaction phase where war-related scenarios were alternated with natural/neutral scenarios (recovery scenarios), with the aim to cycle stress reactions to the war-related scenario with recovery reactions to natural or neutral scenarios; and a post-test phase aimed at gathering information about the overall VR experience (whose results are not presented in this paper).

### 2.2. Sample Description

Participants were recruited through referrals, community flyers, and university web listings advertising the possibility to experience VR, and they were subject to a pre-screening procedure. Specifically, participants with a history of trauma related to violent events or armed conflict, or those that hypothesized having disturbing reactions to images linked to war scenarios, were excluded to avoid triggering traumatic memories or emotional discomfort, and to ensure their well-being during the study and the integrity of the study results. A total of 151 Italian individuals aged between 18 and 35 completed the recruitment survey and the final sample consisted of 52 participants, of which 38 were women (73%) with a mean age of 24.83 years, and 14 were men (27%) with a mean age of 25.50 years. Participants assigned to the natural condition formed three Natural groups (Tropical Island *n* = 13, Meadow *n* = 13, and Forest *n* = 12) on the basis of their chosen scenario.

Among the participants, 75% were students, and their previous experience with VR was approximately evenly distributed, with 59.6% having no prior experience and 40.4% having some experience.

### 2.3. Procedure

Eligible participants were contacted to confirm their participation and arrange the experimental session. The experiment was conducted in the IDEaCT Social Lab at the Department of Social and Developmental Psychology, Sapienza University of Rome, and was approved by the Ethics Committee of Sapienza University of Rome (Research ID: 66/2023).

**Figure 1:**
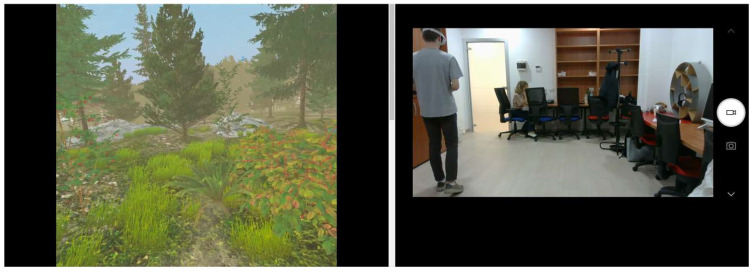
Screenshot of the experimental session.

Upon arriving at the lab, participants were asked to read and sign an informed consent form and were fitted with a Polar H10 HR monitor to track their HR. The researcher then provided them with the Oculus Quest 2, a standalone VR system that includes a headset (HMD) and two controllers, and explained how to use the device. At this point (pre-test phase), the experimental session recording began, capturing both the participants’ behavior in the room and the VR system’s mirrored display (see Figure 1).

Participants were also instructed on how to perform a controlled breathing technique, which they were advised to use if their physiological data showed elevated stress levels during the session.

After the pre-test phase, participants entered the baseline phase and were instructed to select and explore each of the three natural available scenarios, freely moving within the virtual environment and observing their surroundings.

They first explored the natural scenarios in the following order: Tropical Island, Meadow, and Forest. After spending 2 min in each scenario, participants were prompted to press a button, where self-report emotional measures were embedded, which they directly completed within the VR environment.

After exploring and evaluating all three natural scenarios, participants were asked to select their favorite natural scenario, which would serve as a “comfort zone” for the next phase of the experiment. This user-centered approach [47] was used since prior literature emphasizes that congruence between environmental preference and environment type, as well as compatibility with the individual’s inclinations and interpretations [24], can influence people’s judgments of potential restoration [48]. Moreover, since research has shown that preferred places are associated with a greater perception of the site’s restorative potential [49], participants were allowed to choose their favorite scenarios from three of the most commonly preferred natural environment types, with the aim of potentially maximizing their restorative benefits.

Next, participants were introduced to the 9 war-related scenarios—alternated with a recovery scenario (the chosen natural or neutral scenario)—in the following order: Party, War shelter, Medical Camp, Wreckage, Mass graves, Crossfire, Hostages, Guerrilla, and Gunpoint. Participants entered the first viewpoint of each scenario and, in some cases (Party, Wreckage, Mass graves, Crossfire, Guerrilla) could move through teleportation to other viewpoints by selecting travel points within the scenario.

After each war-related scenario and each recovery scenario, participants were required to assess their emotional states.

Following each war-related scenario, participants returned to their recovery scenario for about two minutes.

**Figure 2:**
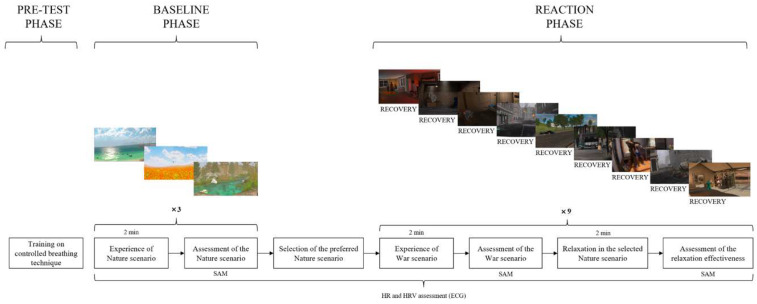
Schematic experimental procedure adopted for the study. Note. The post-test assessment phase is not represented in this figure.

The main VR experimental procedure is visually represented in Figure 2.

### 2.4. Virtual Scenarios

For this study, twelve virtual scenarios were created following ecological principles for VR design, comprising three natural environments and nine war-related situations.

**Figure 3:**
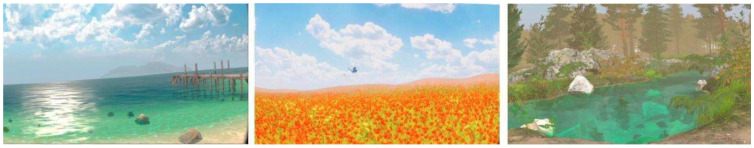
Representative view of the natural scenarios within Virtual Reality (in order: “Tropical Island”, “Meadow”, “Forest”).

The natural scenarios (Figure 3) were developed following the eight components of the Biophilic Effect [49], which include sunlight, color, gravity, fractal elements, curves, details, water, and life. These elements are known to promote relaxation and stress recovery, offering a healing effect on the human body. Specifically, the three natural scenarios analyzed in this study represented a tropical island, a meadow, and a forest. Building on Salingaros’ framework [50], these scenarios were evaluated by the entire group of authors, who assigned a score to each biophilic component, as follows: 0 = absent, 1 = low, 2 = medium, and 3 = high. In cases of divergence, the results were discussed among the authors to reach a consensus on the evaluation. This process allowed us to categorize the environments based on their biophilic effects. Table S1 (see Supplementary Materials) presents the scores assigned by the authors to the three natural environments. The results indicate that the Meadow received the lowest overall score, while the Tropical Island and Forest scenarios achieved similar, higher scores.

**Figure 4:**
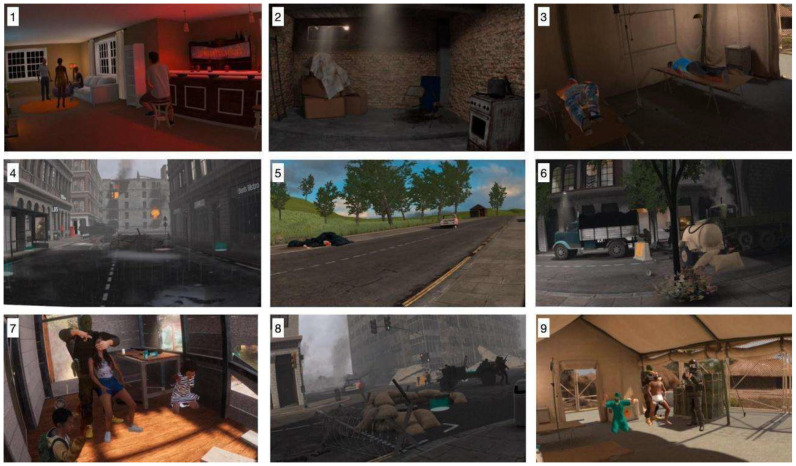
Representative view of the war-related scenarios within Virtual Reality (in order: “Party”, “Shelter”, “Medical camp”, “Wreckage”, “Mass graves”, “Crossfire”, “Hostages”, “Guerrilla”, “Gunpoint”).

On the other hand, representative views of the nine war-related scenarios are shown in Figure 4. The first of these scenarios, “Party” (1), was included as a “trigger” scenario due to its sound and light features which may evoke arousing responses. The remaining eight scenarios depict various aspects of armed conflicts (Figure 4), including (2) war shelter, (3) a medical camp, (4) wreckage, (5) mass graves, (6) crossfire, (7) hostages, (8) guerrilla, and (9) gunpoint situations. These war-related scenarios vary in terms of stress induction and activation triggers, encompassing different locations, actions, social presence, and soundscapes. These scenarios were created to compare the physiological and psychological effects of war scenes with those of natural representations. According to previous literature [51,52], war scenarios are stress inducers. In particular, certain elements, such as seeing dead bodies or discovering human remains, not being able to help sick or wounded civilians, and seeing destroyed houses and villages, are reported as stressors by military operators [53], and have been considered for the construction of our war scenarios.

### 2.5. Measures

In the recruitment survey, participants were asked to complete a number of questions concerning socio-demographic indicators (i.e., gender, age, highest level of educational achievement, country of birth, employment status), prior experience with video-games and VR (frequency of use and types of games), and their level of connection with nature.

Screening questions addressed war-related traumas and anticipated negative reactions to war-related content. Participants were queried about personal war traumas or memories related to armed conflict (yes/no). Additionally, they reported the disturbance level experienced when witnessing war scenes through various media and anticipated discomfort in a virtual reality war simulation (rated from 1 = not at all to 5 = very much). If a participant reported war traumas and indicated high anticipated discomfort (4 = quite a bit, or 5 = very much), they were excluded from the study.

**Figure 5:**
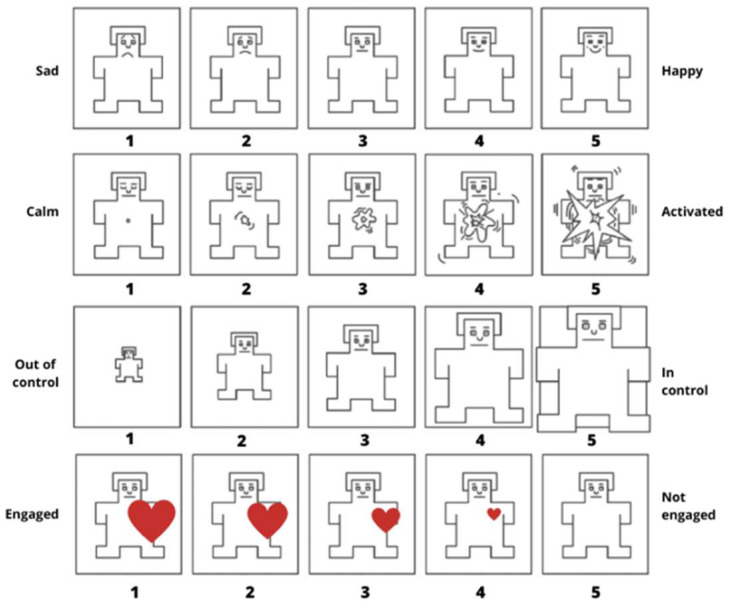
Self-Assessment Manikin scale for the assessment of valence, arousal, control, and engagement used in VR. Note. English translation from the Italian version used in the study.

To measure participants’ emotional state after the experience of the VR scenario, an adapted version of the Self-Assessment Manikin scale [54] was adopted. The SAM is a pictorial scale that is widely used for the assessment of emotional states, in terms of valence (happy—sad), arousal (calm—activated) and in control (out of control—in control). A further dimension was added in the scale to assess the degree of engagement in the VR scenario (engaged—not engaged). The engagement items were adjusted by reverse-coding to ensure consistent interpretation across the scale. The four components will be considered separately in the analyses. Participants were asked to indicate their emotions on the 5-point rating scale (see Figure 5).

To measure physiological reactions to both natural and war-related scenarios, the Polar H10 heart rate (HR) monitor was used to capture participants’ HR and HRV during the experimental session, as an indicator of emotional reactions and recovery from activation. All HRV analyses were conducted using the Kubios HRV Premium software (version 3.3.1). Among the various HRV metrics, the Root Mean Square of Successive Differences (RMSSD), a time-domain HRV metric, is particularly sensitive to Autonomous Nervous System (ANS) activity, and it is an excellent indicator of short-term HRV and individual emotional reactions. More specifically, Mean HR and RMSSD were considered as direct indicators of cardiac activity. Higher HR values are associated with a higher emotional activation [55]. In contrast, higher RMSSD values mean a lower activation, and vice versa [56]. For the physiological data analysis, full data were available only for 44 subjects (Neutral group *n* = 14, Forest *n* = 11, Meadow *n* = 10, and Tropical Island *n* = 9), since data from 8 subjects were unusable due to technical issues encountered during data collection.

### 2.6. Analytic Strategies

Descriptive analyses were conducted to examine the demographic characteristics of participants, as well as the scores for virtual scenarios in terms of participants’ emotional responses.

For data available at the baseline phase, to ensure that the four participant groups (three Natural groups exposed to natural scenarios and one Neutral group exposed to a neutral scenario viewed after the war-related scenario) were equivalent in terms of the ECG physiological parameters at baseline (Mean HR and HRV), a series of ANOVAs were conducted with a between-subjects factor representing the four groups.

For the baseline phase, we performed a series of ANOVAs with the three types of natural scenarios to assess as repeated factors (Tropical Island, Meadow, and Forest) and group conditions as between factors (Neutral group vs. Tropical Island group vs. Meadow group vs. Forest group). The aim of this analysis was to confirm that there are no differences among all natural virtual scenarios in the four components of the SAM (valence, arousal, control, and engagement) and psychological reactions, regardless of the Natural group, and to assess differences among natural scenarios in terms of perceived emotional and physiological responses. Further, preliminary analyses were conducted to assess the realism, presence, and usability of the virtual scenarios, with results reported in Tables S2 and S3 (see Supplementary Materials).

Additionally, data in the reaction phase have been used to verify our research hypothesis (H1 and H2), a series of mixed-design ANOVAs with a cycle factor with two repeated conditions (pre- and post-exposure to recovery scenarios), a type of cycle factor with nine repeated conditions (the nine war-related scenarios cycled with recovery scenario) and a group of conditions for between-factor (Neutral group vs. Tropical Island group vs. Meadow group vs. Forest group) was performed to investigate changes in SAM scores and HR. HRV measures were taken before and after exposure to natural (as opposed to neutral) virtual scenarios explored following the nine war-related virtual scenarios in the four groups. This analysis evaluated whether the exploration of natural environments generated a restorative experience and restored emotional states. In particular, we were interested in the following effects: the main effect of group conditions used to verify differences between the VR natural scenario and VR neutral scenario (H1); the interaction between the type of cycle and the cycle factors, to test the recovery hypothesis (H2) and the variation of the recovery effect as a function of the war-related scenario; finally, the interaction between the cycle factor and the group factor to test whether the recovery effect is present when participants have the natural scenario as their recovery scenario, and not when they have a neutral scenario (H2). For these interaction effects, a multivariate simple-effect post-hoc analysis has been performed to check for which pair of conditions we found significant differences.

Finally, using data from the baseline and reaction phases, we tested our last research hypothesis (H3) by performing a series of mixed-design ANOVAs with a time factor contrasting perceived emotions and physiological responses from the baseline phase and the recovery responses taken from reaction phase (considering the average across the nine recovery scenarios), as well as group conditions as a between factor (Neutral group vs. Tropical Island group vs. Meadow group vs. Forest group). The interaction effect between the time factor and the group conditions factor has been used to verify whether the perceived emotional responses and physiological reactions before and after being exposed to war-related scenarios are the same for participants who have the natural scenarios as recovery scenarios, and different for those who have the neutral scenario.

## 3. Results

### 3.1. Baseline Phase: Participants’ HR and RMSSD (HRV) Equivalence Baselines

Non-significant differences emerged for the Mean HR index, *F*(3, 142) = 0.430, *p* = 0.732, *η^2^* = 0.031), and for the RMSSD (HRV) index, *F*(3, 17,433) = 0.947, *p* = 0.427, *η^2^* = 0.066); the four participant groups were equivalent in terms of physiological activation.

### 3.2. Baseline Phase: Preliminary Analysis of Natural Scenarios on SAM Components

The analysis revealed no significant main effects of the type of natural scenario or group factor on valence, arousal, or control, though a significant interaction was found for valence (scenario × group, *p* = 0.002), particularly between the Forest and Meadow groups, when evaluating the Forest scenario (*p* = 0.048). Regarding engagement, a significant main effect of the type of natural scenario (*p* = 0.003) and an interaction with the group (*p* = 0.021) were observed, indicating differences in engagement across scenarios and Natural groups. These results indicate that, overall, participants did not exhibit relevant differences in emotional responses across the different types of natural scenarios or between the groups.

### 3.3. Baseline Phase: Preliminary Analysis of Natural Scenarios on HR and RMSSD (HRV)

For Mean HR, the main effects of the types of natural scenarios were significant (*F*(2, 39) = 5.043, *p* = 0.011, *η^2^_P_* = 0.205), while the effect of group was not (*F*(3, 1121) = 1.460, *p* = 0.240, *η^2^_P_* = 0.099). Similarly, the interaction between type of scenario and group factors was not significant—*F*(6, 80) = 0.386, *p* = 0.886, *η^2^_P_* = 0.028. These findings suggest that while Mean HR was influenced by the type of natural scenario, neither the group factor alone nor its interaction with the type of natural scenario resulted in significant differences in physiological arousal levels.

For RMSSD, no significant main effects were found for either the type of natural scenario (*F*(2, 39) = 0.805, *p* = 0.454, *η^2^_P_* = 0.040) or the group (*F*(3, 6569) = 1.038, *p* = 0.386, *η^2^_P_* = 0.072). Additionally, the interaction between the type of scenario and group was not significant (*F*(6, 80) = 0.793, *p* = 0.578, *η^2^_P_* = 0.056). These results indicate that participants’ HRV did not vary significantly based on the type of natural scenario or across the groups.

### 3.4. Reaction Phase: Analysis of the Differences Between the Natural and Neutral Groups (H1–H2)

#### 3.4.1. Reaction Phase: Analysis of Valence of Perceived Emotions (SAM) as a Function of Groups and Post-War Recovery Scenarios (H1a–H2a)

Table S4 (see Supplementary Materials) shows descriptive statistics of valence scores in war-related and post-war-related recovery scenarios across groups. Overall, groups who recovered in natural virtual environments consistently showed higher mean valence scores compared to the Neutral group, indicating a more positive emotional state in natural virtual settings experienced after the exposure to war-related scenarios, compared to the Neutral group.

**Figure 6:**
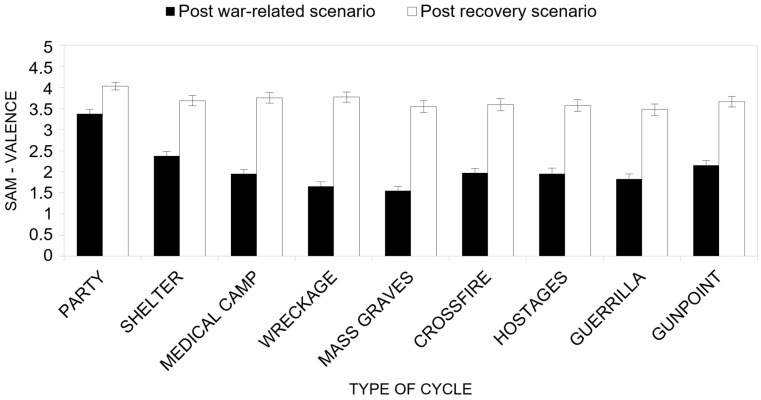
Perceived valence (SAM) as a function of the type of cycle factor and the cycle factor.

Concerning the first research hypothesis (H1a), the results from ANOVA (Figure 6) reveal a near-significant main effect of the group (*F*(3, 46) = 2.706, *p* = 0.056, *η^2^_p_* = 0.150), with participants with natural scenarios as their recovery scenario reporting higher valence ratings than those in the Neutral group. More specifically, participants in the Neutral group reported lower emotional valence (*M* = 2.57) compared to those in the Forest (*M* = 2.92), Meadow (*M* = 3.03), and Tropical Island (*M* = 3.03) groups. These findings suggest that the Neutral group experienced less positive emotional responses in the post-war recovery scenarios compared to the other Natural groups, which were associated with virtual natural environments.

Moreover, a significant main effect of the type of cycle factor was also observed on emotional valence scores (*F*(8, 39) = 14.14, *p* < 0.001, *η^2^_P_* = 0.744), meaning that overall, the average valence between the war-related scenario experienced before the recovery scenarios and the subsequent emotional valence in the recovery changed among war-related scenarios (respectively, Party *M* = 3.70; Shelter *M* = 3.03; Medical camp *M* = 2.86; Wreckage *M* = 2.72; Mass graves *M* = 2.55; Crossfire *M* = 2.78; Hostages *M* = 2.77; Guerrilla *M* = 2.65; Gunpoint *M* = 2.91).

The main effect of the cycle factor was also significant (*F*(1, 46) = 190.29, *p* < 0.001, *η^2^_P_* = 0.805), meaning that, overall, the average perceived valence after the war-related scenario was lower (*M* = 2.09) than that after the subsequent recovery scenarios (*M* = 3.68).

Turning to the research hypothesis concerning the recovery effect (H2a), the interaction between the type of cycle factor and the cycle factors was significant (*F*(8, 39) = 8,81, *p* < 0.001, *η^2^_P_* = 0.644). In particular, it was found that, overall, the average of the perceived valence after the war-related scenario was lower than that after the recovery scenario for all war-related scenarios (Figure 6), meaning that, on average, the recovery scenario always increased the average valence of experienced emotions. The smallest difference was observed for the Party scenario (post-war-related scenario *M* = 3.38; post-recovery scenario *M* = 4.03), while the highest difference was observed for the Mass graves scenario (post-war-related scenario *M* = 1.55; post recovery scenario *M* = 3.55).

**Figure 7:**
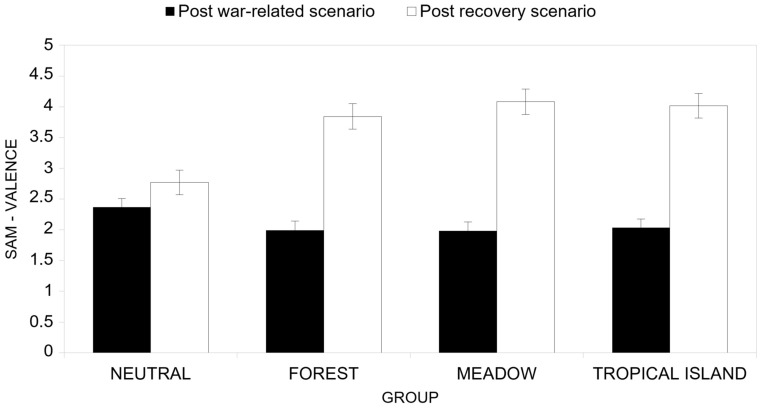
Interaction effect between the cycle factor and the group factor in terms of valence.

Also, the interaction effect between the cycle factor and the group conditions factor (Figure 7) was significant (*F*(3, 46) = 12,30, *p* < 0.001, *η^2^_P_* = 0.445). In particular, we found that for the Neutral group, the difference between the perceived valence after the war-related scenario and the perceived valence after the recovery scenario was not significant (*F*(1, 46) = 3.19, *p* = 0.081, *η^2^_P_* = 0.065; post-war scenario *M* = 2.37; post-recovery scenario *M* = 2.77), while the difference was significant for the Forest group (*F*(1, 46) = 62.47, *p* < 0.001, *η^2^_P_* = 0.576; post-war scenario *M* = 1.99; post-recovery scenario *M* = 3.84), for the Meadow group (*F*(1, 46) = 80.48, *p* < 0.001, *η^2^_P_* = 0.636; post-war scenario *M* = 1.98; post-recovery scenario *M* = 4.08) and for the Tropical Island group (*F*(1, 46) = 77.60, *p* < 0.001, *η^2^_P_* = 0.628; post-war scenario *M* = 2.03; post-recovery scenario *M* = 4.02).

#### 3.4.2. Reaction Phase: Analysis of Arousal of Perceived Emotion (SAM) as a Function of Groups and Post-War Recovery Scenarios (H1b–H2b)

Descriptive statistics of arousal scores in war and post-war recovery scenarios across groups are presented in Table S5 (see Supplementary Materials). Overall, participants in the Neutral group exhibited consistently higher arousal mean scores compared to those in the Natural groups exposed to natural scenarios, indicating a higher activation state in neutral settings experienced after the exposure to war-related scenarios.

**Figure 8:**
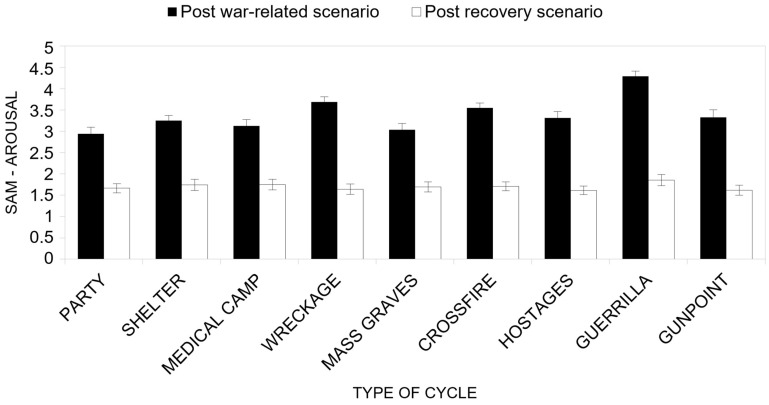
Perceived arousal (SAM) as a function of the type of cycle factor and the cycle factor.

Concerning the first research hypothesis (H1b; Figure 8), the results from ANOVA reveal that the main effect of the group on arousal ratings was not significant (*F*(3, 46) = 2.196, *p* = 0.101, *η^2^_P_* = 0.125). However, by inspecting the mean scores, it can be observed that, overall, the Neutral group reported the highest arousal estimates (*M* = 2.79), while the Natural groups scored lower estimates (Forest group *M* = 2.39; Meadow group *M* = 2.33; Tropical Island group *M* = 2.67).

On the other hand, a significant main effect of the type of cycle factor on arousal scores (*F*(8, 39) = 12.24, *p* < 0.001, *η^2^_P_* = 0.677) was found, thus suggesting that, overall, the average participants’ perceived arousal differed significantly among the types of war-related scenarios (respectively, Party *M* = 2.30; Shelter *M* = 2.50; Medical camp *M* = 2.47; Wreckage *M* = 2.66; Mass graves *M* = 2.37; Crossfire *M* = 2.63; Hostages *M* = 2.46; Guerrilla *M* = 3.07; Gunpoint *M* = 2.47). Also, the main effect of the cycle factor proved significant (*F*(1, 46) = 227.66, *p* < 0.001, *η^2^_P_* = 0.832), implying that the average perceived arousal of experienced emotion after the war-related scenario (*M* = 3.39) was significantly higher than that after the recovery scenario (*M* = 1.70). In general, the recovery scenarios were effective in reducing the arousal induced during the war-related scenario.

Considering the interaction effect of the type of the cycle factor with the Cycle Factor (H2b), a significant effect (*F*(8, 39) = 6.95, *p* < 0.001, *η^2^_P_* = 0.588) is found. By performing multivariate simple effect post-tests, we found that, overall, the difference between perceived arousal after the war-related scenario and after the recovery scenario was significant for all types of war scenarios (Figure 8). Overall, this result supports the effectiveness of the recovery effect of the recovery scenario after the stressing war-related scenario.

**Figure 9:**
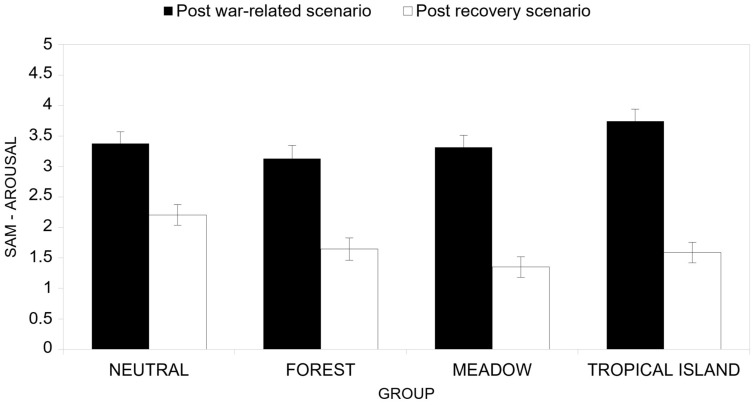
Interaction between the cycle factor and the group factor in terms of arousal.

Finally, the interaction effect of the cycle factor and the group conditions factor (H2, Figure 9) was proven significant (*F*(3, 46) = 4.12, *p* < 0.001, *η^2^_P_* = 0.212). Post-hoc tests indicated that, differently from the valence measure, all groups of participants reported a significant difference between their perceived arousal after the war-related scenario and the arousal perceived after the recovery scenario. It is worth nothing that the Neutral group reported the least difference (post-war-related scenario *M* = 3.38; recovery scenario *M* = 2.21), while the Tropical Island group reported the highest difference (post-war-related scenario *M* = 3.74; recovery scenario *M* = 1.59).

#### 3.4.3. Reaction Phase: Physiological Reactions as a Function of Groups and Post-War Recovery Scenarios (H1c–H2c)

##### Mean HR

Descriptive statistics of Mean HR scores in war and post-war recovery scenarios across groups are presented in Table S6 (see Supplementary Materials). Overall, participants in the Neutral and Forest groups consistently displayed lower mean HR scores across most post-war recovery scenarios compared to the Meadow and Tropical Island groups, suggesting lower physiological activation.

**Figure 10:**
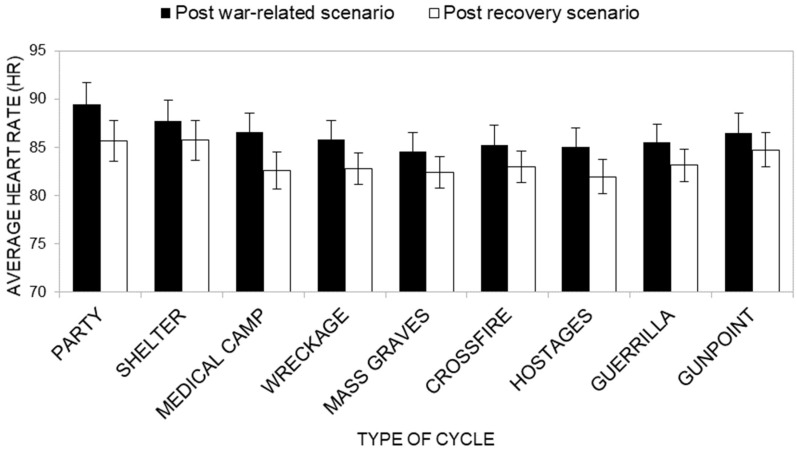
Mean HR as a function of the type of cycle factor and of the cycle factor.

Concerning the first research hypothesis (H1c; Figure 10), the ANOVA results do not show a significant main effect of the group factor on Mean HR (*F*(3, 40) = 2.01, *p* = 0.128, *η^2^_P_* = 0.121). Inspecting average HR scores, the Neutral group reported, overall, an average HR of *M* = 82, while the Forest group had an average HR of *M* = 80, the Meadow group an average HR of *M* = 91, and finally the Tropical Island group had an average HR of *M* = 86.

On the other hand, the findings show a significant main effect of the type of cycle factor on Mean HR (*F*(8, 33) = 5.76, *p* < 0.001, *η^2^_P_* = *0.583*), suggesting that the type of war scenario participants experienced influenced participants’ average HR during the war-related scenario and recovery scenario (respectively, Party *M* = 88; Shelter M = 87; Medical camp *M* = 85; Wreckage *M* = 84; Mass graves *M* = 83; Crossfire *M* = 84; Hostages *M* = 83; Guerrilla *M* = 84; Gunpoint *M* = 86).

Also, the main effect of the cycle factor was significant (*F*(1, 40) = 16.81, *p* < 0.001, *η^2^_P_* = *0.296*), with the average HR recorded during the war-related scenario being higher (*M* = 86) than that recorded during the recovery scenario phase (*M* = 84). This result shows that, in general, the recovery scenarios were effective in determining a recovery effect.

Contrary to our hypotheses (H2c), the interaction effect of the type of the cycle Factor and the cycle factor (Figure 10) was not significant (*F*(8, 33) = 0.846, *p* = 0.570, *η^2^_P_* = *0.170*). However, multivariate post-hoc tests showed that the difference between the average HR values during the war-related scenario (*M* = 89) and the average HR during the recovery scenario (*M* = 86) was significant, and in the expected direction, for the Party (*F* (1, 40) = 9.33, *p* = 0.004, *η^2^_P_* = *0.189*), Medical Camp (*F* (1, 40) = 20.33, *p* < 0.001, *η^2^_P_* = *0.337*; average HR during the war-related scenario *M* = 87; average HR during the recovery scenario *M* = 83), Wreckage (*F* (1, 40) = 10.77, *p* = 0.002, *η^2^_P_* = *0.212*; average HR during the war-related scenario *M* = 86; average HR during the recovery scenario *M* = 83), Crossfire (*F* (1, 40) = 5.44, *p* = 0.025, *η^2^_P_* = *0.120*; average HR during the war-related scenario *M* = 85; average HR during the recovery scenario *M* = 83), Hostage (*F* (1, 40) = 7.24, *p* = 0.010, *η^2^_P_* = *0.153*; average HR during the war-related scenario *M* = 85; average HR during the recovery scenario *M* = 82) and Guerrilla scenarios (*F* (1, 40) = 4.38, *p* = 0.043, *η^2^_P_* = *0.099*; average HR during the war-related scenario *M* = 85; average HR during the recovery scenario *M* = 83), but not for the Shelter (*F* (1, 40) = 3.85, *p* = 0.057, *η^2^_P_* = *0.088*; average HR during the war-related scenario *M* = 88; average HR during the recovery scenario *M* = 86), Mass Grave (*F* (1, 40) = 3.11, *p* = 0.086, *η^2^_P_* = *0.072*; average HR during the war-related scenario *M* = 85; average HR during the recovery scenario *M* = 82), or Gunpoint scenarios (*F* (1, 40) = 2.42, *p* = 0.128, *η^2^_P_* = *0.057*; average HR during the war-related scenario *M* = 86; average HR during the recovery scenario Time *M* = 85).

**Figure 11:**
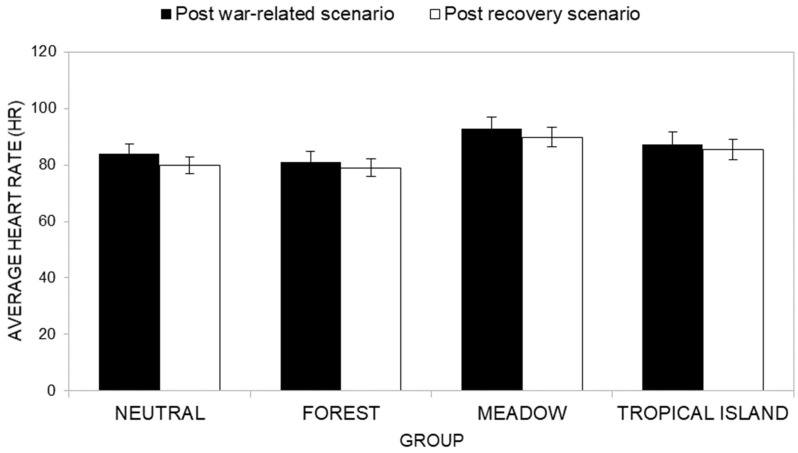
Interaction between the cycle factor and the group factor in terms of physiological arousal (Mean HR).

Finally, the interaction effect of the cycle factor and group conditions factor (Figure 11) was also not significant (*F* (3, 40) = 0.77, *p* = 0.518, *η^2^_P_* = *0.055*). Multivariate post-hoc analysis showed that for the Neutral group, the average HR recorded during the war-related scenario (M = 84) was significantly higher than the average HR (*M* = 80) recorded during the recovery scenario (*F* (1, 40) = 10.77, *p* = 0.002, *η^2^_P_* = *0.212*). A similar pattern was also observed for the Meadow group (*F* (1, 40) = 4.48, *p* = 0.041, *η^2^_P_* = *0.101*; average HR during the war-related scenario *M* = 93; average HR during the recovery scenario Time *M* = 90). For the Forest group (*F* (1, 40) = 1.18, *p* = 0.148, *η^2^_P_* = *0.052*; average HR during the war-related scenario *M* = 81; average HR during the recovery scenario *M* = 79) and for the Tropical Island group (*F* (1, 40) = 1.64, *p* = 0.208, *η^2^_P_* = *0.039*; average HR during the war-related scenario *M* = 87; average HR during the recovery scenario *M* = 85), the difference was not significant.

##### RMSSD (HRV)

Descriptive statistics of RMSSD scores in war and post-war recovery scenarios across groups are presented in Table S7 (see Supplementary Materials). Overall, participants in the Neutral group showed consistently higher average RMSSD values across most of the recovery scenarios compared to the Natural groups. Higher RMSSD values indicate greater HRV, suggesting that the Neutral group was more physiologically flexible and more able to adapt to the stressors induced by the war-related scenario. In contrast, the Forest, Meadow, and Tropical Island groups exhibited on average lower RMSSD values, indicating reduced HRV. This lower variability suggests that participants with a Natural scenario as their recovery scenario in general showed less adaptability or recovery capacity in terms of autonomic regulation.

**Figure 12:**
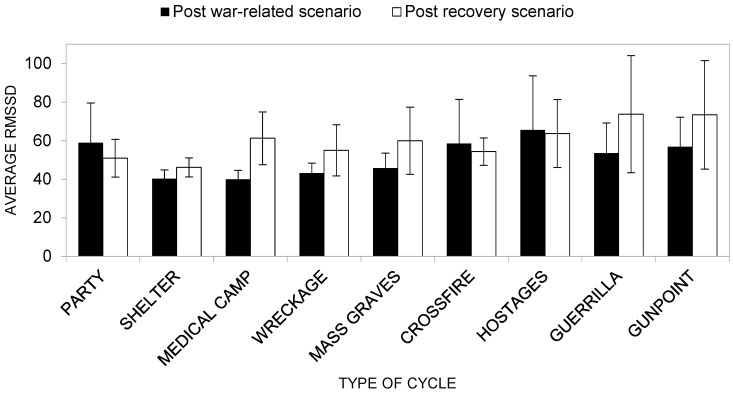
Average RMSSD as a function of the type of cycle factor and of the cycle factor.

Concerning the first research hypothesis (H1c; Figure 12), the ANOVA results reveal a non-significant main effect of the group conditions factor on RMSSD (*F*(3, 40) = 0.898, *p* = 0.451, *η^2^* = 0.063). Upon inspecting the average RMSSD values of the four groups of participants, we find that the Neutral group reported, overall, the highest average RMSSD value (*M* = 88.13), while the groups with natural scenarios reported lower and similar average RMSSD values (respectively, Forest group *M* = 43.31; Meadow group *M* = 48.12; Tropical Island group *M* = 43.97).

Similarly, we found no significant effects on RMSSD of the type of cycle factor (*F* (8, 33) = 0.557, *p* = 0.805, *η^2^* = 0.119). The average RMSSD values ranged from a minimum of *M* = 43.23 for the Wreckage scenario to the maximum of *M* = 65.14 for the Gunpoint scenario. Also, the main effect of the cycle factor was not significant, even if the *p*-value approached the critical value (*F*(1, 40) = 3.70, *p* = 0.062, *η^2^* = 0.085), with the average RMSSD recorded during the war-related scenario lower (*M* = 51.40) than the average RMSSD recorded during the recovery scenario (*M* = 59.86).

Turning to the interaction effect between the type of cycle factor and the cycle factor (H2c), we find that the effect was not significant (*F*(8, 33) = 0.258, *p* = 0.975, *η^2^* = 0.059). In this case, post-hoc analysis showed no significant differences between average RMSSD recorded during the war-related scenario and that recorded during the recovery scenario for any of the war scenarios. However, as can be seen in Figure 12, for the Shelter, Medical camp, Wreckage, Mass grave, Guerrilla and Gunpoint scenarios, the pattern of RMSSD means was in the expected direction, with the average RMSSD recorded during the war-related scenario lower than that recorded during the Recovery scenario. Even if this pattern does not satisfy the statistical criteria, it shows that the recovery scenario played a recovery role during the experiment.

**Figure 13:**
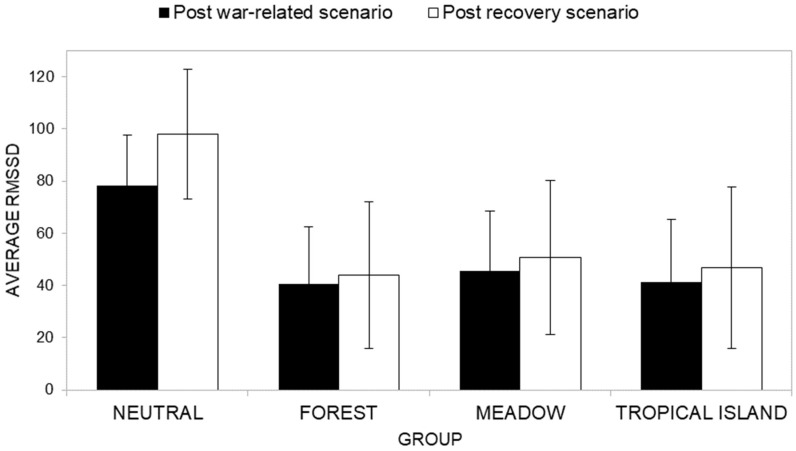
Interaction between the cycle factor and the group factor in terms of physiological arousal (RMSSD).

Finally, the interaction effect between the cycle factor and the group conditions factor (Figure 13) was not significant (*F*(3, 40) = 0.894, *p* = 0.453, *η^2^* = 0.063). Post-hoc analysis showed that in the Neutral group, the average RMSSD recorded during the war-related scenario (*M* = 78.24) was significantly (*F*(1, 40) = 6.62, *p* = 0.014, *η^2^* = 0.142) higher than that recorded during the recovery scenario (*M* = 98.03). On the contrary, significant differences for the Forest group (*F*(1, 40) = 0.164, *p* = 0.688, *η^2^* = 0.004) were found (average RMSSD during the war-related scenario *M* = 40.55; average RMSSD during the recovery scenario *M* = 44.07), as well as for the Meadow group (*F*(1, 40) = 0.164, *p* = 0.688, *η^2^* = 0.008; average RMSSD during the war-related scenario *M* = 45.61; average RMSSD during the recovery scenario *M* = 50.63) and the Tropical Island group (*F*(1, 40) = 0.331, *p* = 0.568, *η^2^* = 0.008; average RMSSD during the war-related scenario *M* = 41.21; average RMSSD during the recovery scenario *M* = 46.73). It is worth noting that the pattern of average RMSSD showed by participants with the Natural scenario as their recovery scenario is congruent with our research hypotheses.

### 3.5. Analysis of the Effectiveness of Natural Scenarios in Restoring the Baseline Values (H3)

#### 3.5.1. Comparison Between Average Valence of Baseline Phase and the Average Valence of Reaction Phase Across the Group Factors (H3a)

Descriptive statistics of valence scores across the group conditions factor at the baseline phase (T1) and at the reaction phase (T2) are presented in Table S4 (see Supplementary Materials). Overall, it can be noticed that participants reported significantly lower valence scores during the baseline phase (T1) compared to the reaction phase (T2).

The ANOVA results reveal a significant main effect of the time factor (*F*(1, 48) = 12.72, *p* < 0.001, *η^2^* = 0.209), with the perceived valence in the baseline phase being significantly higher (*M* = 4.09) than the average perceived valence after the recovery scenario in the reaction phase (*M* = 3.68). This indicates that participants’ valence scores substantially shifted in emotional valence from the baseline phase to the reaction phase.

Nevertheless, the main effect for the group factor was also significant (*F*(3, 48) = 4.98, *p* < 0.004, *η^2^_P_* = 0.237), with the Neutral group reporting an average valence at the baseline phase that was lower (*M* = 3.37) than the average valence evaluated after the recovery scenario in the reaction phase of the Forest group (*M* = 4.03), the Meadow group (*M* = 4.07) and the Tropical Island group (*M* = 4.05).

**Figure 14:**
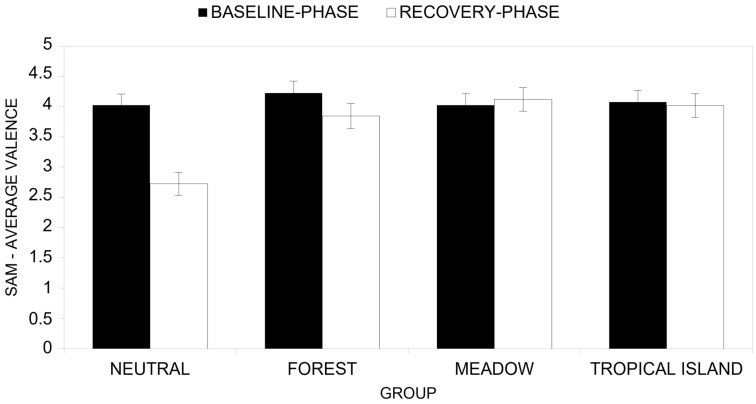
Comparison of T1 (baseline phase) and T2 (reaction phase) across groups in terms of valence (SAM).

A significant effect was also observed for the interaction between time and group factors (Figure 14, *F*(3, 48) = 7.72, *p* < 0.001, *η^2^_P_* = 0.326), showing that the change in valence scores from baseline to reaction phase differed significantly across groups. More specifically, post-hoc analyses showed that the Neutral group reported a significantly (*F*(1, 48) = 34.30, *p* < 0.001, *η^2^_P_* = 0.417) higher perceived valence at baseline (*M* = 4.02) than after the recovery scenario in the reaction phase (*M* = 2.72). On the contrary, the Forest group (*F*(1, 48) = 2.51, *p* = 0.120, *η^2^_P_* = 0.050; perceived valence at baseline *M* = 4.22; perceived valence at recovery scenario in the reaction phase *M* = 3.84), the Meadows group (*F*(1, 48) = 0.17, *p* = 0.685, *η^2^_P_* = 0.003; perceived valence at baseline *M* = 4.03; perceived valence under recovery scenario in the reaction phase *M* = 4.12) and the Tropical Island group (*F*(1, 48) = 0.07, *p* = 0.796, *η^2^_P_* = 0.001; perceived valence at baseline *M* = 4.08; perceived valence under recovery scenario in the reaction phase *M* = 4.02) reported a non-significant difference between baseline phase and reaction phase.

#### 3.5.2. Comparison of the Baseline Phase and Reaction Phase Levels of Arousal Across Groups (H3b)

Descriptive statistics of arousal scores across groups for the baseline phase (T1) and the reaction phase (T2) are presented in Table S5 (see Supplementary Materials). Overall, participants consistently reported higher arousal levels during the baseline phase (T1) compared to the reaction phase (T2).

The ANOVA results reveal a significant main effect of time (*F*(1, 48) = 4.70, *p* = 0.035, *η^2^_P_* = 0.089), indicating that participants experienced significantly higher levels of arousal at the baseline phase (T1) compared to the reaction phase (T2).

A significant main effect was found for the group factor (*F*(3, 48) = 2.82, *p* = 0.049, *η^2^_P_* = 0.150), with the mean arousal scores for the Neutral group being higher (*M* = 2.11) than those reported by the Meadow group (*M* = 1.509), but they were not different in terms of average arousal reported by the Forest group (*M* = 1.80) and the Tropical Island group (*M* = 1.97).

**Figure 15:**
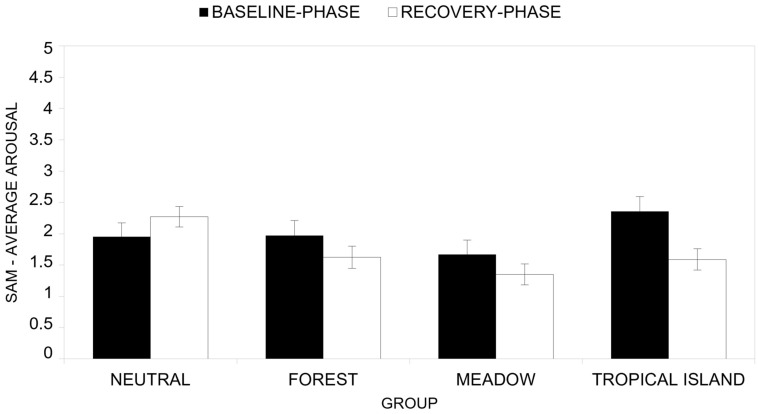
Comparison of T1 (baseline phase) and T2 (reaction phase) across groups in terms of arousal (SAM).

Finally, a significant interaction effect between time and group was observed (Figure 15, *F*(3, 48) = 3.18, *p* = 0.032, *η^2^_P_* = 0.166). Post-hoc analyses of the interaction showed that only the Tropical Island group reported a significantly (*F*(1, 48) = 8.96, *p* = 0.004, *η^2^_P_* = 0.157) higher arousal level at the baseline phase (*M* = 2.36) than at the reaction phase (*M* = 1.59). On the contrary, the Neutral group (*F*(1, 48) = 1.65, *p* = 0.205, *η^2^_P_* = 0.033; arousal level at the baseline phase *M* = 1.95; arousal level at the reaction phase *M* = 2.27), the Forest group (*F*(1, 48) = 1.70, *p* = 0.199, *η^2^_P_* = 0.034; arousal level at the baseline phase *M* = 1.97; arousal level at the reaction phase *M* = 1.62) and the Meadow group (*F*(1, 48) = 1.52, *p* = 0.224, *η^2^_P_* = 0.031; arousal level at the baseline phase *M* = 1.67; arousal level at the reaction phase *M* = 1.35) did not report any significant differences between baseline phase and the reaction phase.

#### 3.5.3. Comparison of T1 (Baseline Phase) and T2 (Reaction Phase) Across Groups in Terms of Physiological Arousal (H3c)

##### Mean HR

Descriptive statistics of Mean HR across groups at the baseline phase (T1) and the reaction phase (T2) are presented in Table S6 (see Supplementary Materials). Across all groups, participants consistently exhibited higher Mean HR scores during the baseline phase compared to the reaction phase.

The ANOVA results reveal a significant main effect of time (*F*(1, 40) = 18.76, *p* < 0.001, *η^2^_P_* = 0.319), indicating that participants, overall, exhibited significantly lower Mean HR scores during the reaction phase (T2, *M* = 86) compared to the baseline phase (T1, *M* = 90).

However, no significant main effect of group was found, *F*(3, 40) = 1.80, *p* = 0.163, *η^2^_P_* = 0.119, indicating that Mean HR scores did not differ significantly across groups (Neutral group HR *M* = 85; Forest group HR *M* = 80; Meadow group HR *M* = 92; Tropical Island group HR *M* = 90).

**Figure 16:**
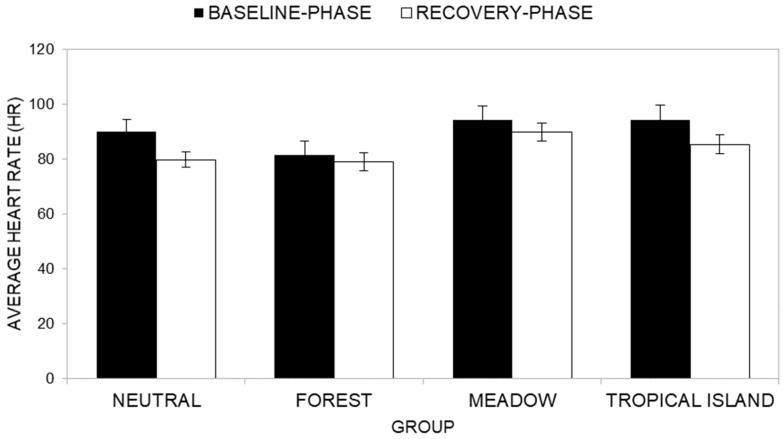
Comparison of average HR at T1 (baseline phase) and at T2 (reaction phase) across groups.

Additionally, the interaction effect between time and group (Figure 16) was not significant (*F*(3, 40) = 1.54, *p* = 0.219, *η^2^_P_* = 0.104). Post-hoc analysis showed that the Neutral group (*F*(1, 40) = 14.91, *p* < 0.001, *η^2^_P_* = 0.272; average HR at baseline phase *M* = 90; average HR at the reaction phase *M* = 80) and the Tropical Island group (*F*(1, 40) = 7.35, *p* = 0.010, *η^2^_P_* = 0.155; average HR at baseline phase *M* = 94; average HR at the reaction phase *M* = 85) reported higher HR values at the baseline phase than at the reaction phase. On the contrary, the Forest group (*F*(1, 40) = 0.78, *p* = 0.384, *η^2^_P_* = 0.019; average HR at baseline phase *M* = 82; average HR at the reaction phase *M* = 79) and the Meadow group (*F*(1, 40) = 1.99, *p* = 0.166, *η^2^_P_* = 0.047; average HR at baseline phase *M* = 94; average HR at the reaction phase *M* = 90) reported non-significant differences between average HR values at the baseline phase and at the reaction phase.

##### RMSSD (HRV)

Descriptive statistics of RMSSD across groups at the baseline phase (T1) and at the reaction phase (T2) are presented in Table S7 (see Supplementary Materials). Across all groups, participants generally exhibited lower RMSSD scores during the baseline phase compared to the reaction phase. This suggests that participants experienced heightened physiological activation during the reaction phase than in the baseline phase.

The ANOVA results do not show a significant main effect of time (*F*(1, 40) = 1.06, *p* = 0.310, *η^2^_P_* = 0.026), indicating that participants exhibited similar levels of RMSSD between the baseline phase (T1, *M* = 46.32) and the reaction phase (T2, *M* = 59.86). Similarly, no significant main effect of the group condition factor was observed (*F*(3, 40) = 0.431, *p* = 0.732, *η^2^_P_* = 0.031), indicating that RMSSD levels did not differ significantly across the groups (respectively, Neutral group RMSSD *M* = 66.40; Forest group RMSSD *M* = 43.30; Meadow group RMSSD *M* = 58.87; Tropical Island group RMSSD *M* = 43.80).

**Figure 17:**
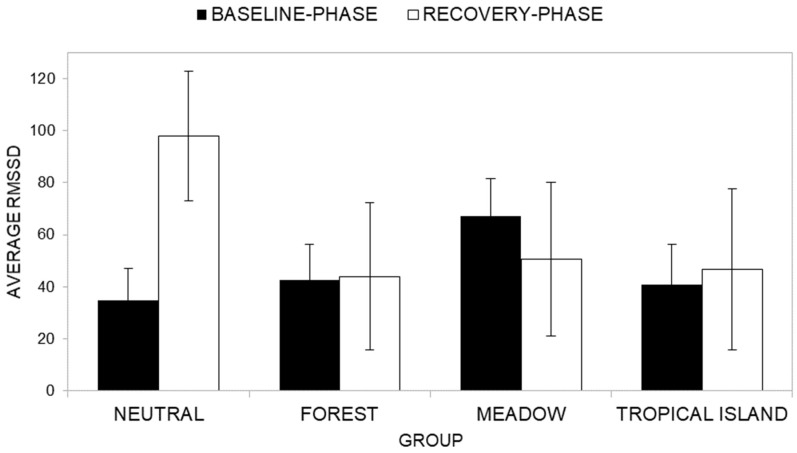
Comparison of T1 (baseline phase) and T2 (reaction phase) across groups in terms of RMSSD.

Furthermore, the interaction effect between time and group (Figure 17) was also not significant (*F*(3, 40) = 2.02, *p* = 0.127, *η^2^_P_* = 0.131). Post-hoc analysis showed that the Neutral group reported a significantly (*F*(1, 40) = 7.55, *p* = 0.009, *η^2^_P_* = 0.159) lower average RMSSD level at the baseline phase (*M* = 34.78) than at the reaction phase (*M* = 98.03). All the other groups reported a non-significant difference between average RMSSD at the baseline phase and at the reaction phase (respectively, Forest group—F (1, 40) = 0.004, *p* = 0.953, *η^2^_P_* = 0.000; average RMSSD at the baseline phase *M* = 42.53; average RMSSD at the reaction phase *M* = 44.07; Meadow group—F (1, 40) = 0.37, *p* = 0.548, *η^2^_P_* = 0.009; average RMSSD at the baseline phase *M* = 67.11; average RMSSD at the reaction phase *M* = 50.62; Tropical Island group—F (1, 40) = 0.042, *p* = 0.839, *η^2^_P_* = 0.001; average RMSSD at the baseline phase *M* = 40.86; average RMSSD at the reaction phase *M* = 46.73).

## 4. Discussion

This experimental study employed a mixed design to examine the emotional and physiological effects of natural versus neutral recovery scenarios following exposure to war-related stressors. Specifically, the research aimed to test whether natural scenarios enhance positive emotions and reduce arousal more effectively than neutral scenarios across different types of war-related stressors.

The first hypothesis (H1) examined differences between Natural and Neutral groups in emotional valence, arousal, and physiological arousal, expecting higher positive emotions and lower emotional and physiological arousal in participants exposed to VR natural scenarios. The results partially support these hypotheses. H1a was supported, as participants recovering in virtual natural scenarios reported higher emotional valence than those in the Neutral group, suggesting that nature-based VR settings promote more positive emotional states. This effect was observed consistently across all three Natural groups. Regarding H1b, while no significant effect of the group on perceived arousal ratings was found, the mean scores suggest a trend in the expected direction, with the Neutral group exhibiting the highest arousal levels. This pattern indicates that participants exposed to the neutral scenario maintained a higher activation state, whereas those in the Natural groups showed relatively lower arousal. Finally, H1c was not supported, as no significant differences in physiological arousal were found between groups. Mean HR was slightly lower in the Forest and Neutral groups, while RMSSD (HRV) values were higher in the Neutral group, suggesting greater physiological flexibility. Conversely, participants in the Natural groups exhibited lower HRV, indicating reduced autonomic recovery, contradicting our hypothesis.

The second hypothesis (H2) explored changes across the cycle (arousing vs. recovery phases) between Natural and Neutral groups in emotional valence and perceived and physiological arousal. We expected that natural scenarios would enhance recovery by increasing positive valence and reducing both perceived and physiological arousal after exposure to arousing stimuli (war-related scenarios). H2 was partially confirmed. The results support significant changes in perceived valence and arousal across the cycles and groups. Specifically, H2a was supported, as participants exposed to natural environments showed a significant increase in positive valence in the recovery phase, whereas this effect was absent in the Neutral group. H2b was partially supported, as both natural and neutral scenarios led to a significant reduction in perceived arousal in the recovery phase, contradicting the hypothesis that natural environments would have a stronger calming effect with respect to neutral stimuli. The findings on H2c were more complex. While all groups exhibited a decrease in mean HR during recovery, significant differences between the arousing and recovery phases emerged only for the Neutral and Meadow groups, contradicting our initial expectations. Similarly, while RMSSD (HRV) increased across all groups—suggesting greater physiological adaptation to stress—only the Neutral group exhibited a significant difference across the cycle, again inconsistent with our hypothesis. Therefore, H2c was not supported. However, despite these inconsistencies in physiological measures, the combined analysis of implicit and explicit data suggests that natural environments facilitated an emotional activation appraised as positive. In contrast, the neutral environment, characterized by lower physiological activation, may have been associated with boredom or disengagement. This suggests that physiological arousal in natural environments may reflect a pleasant stimulation rather than a stress response.

The third hypothesis (H3) investigated differences between the baseline and reaction phases. Specifically, we expected that natural environments would facilitate a return to initial baseline levels more effectively than the neutral scenario. This hypothesis was partially supported. From self-reported data, we see that natural scenarios were more effective in restoring baseline values for perceived valence and arousal, supporting H3a and H3b. Indeed, valence levels were higher in the reaction phase than in the baseline phase for all three Natural groups, while the Neutral group showed lower valence levels in the reaction phase. Regarding perceived arousal, only in the Neutral group were arousal levels lower in the reaction phase compared to baseline, whereas Natural groups showed the opposite trend. In the end, H3c was partially supported. Regarding HR, the results were mixed. The hypothesis was confirmed for the Forest and Meadow groups, where no significant difference was observed between the baseline and reaction phases. It was also supported in the Tropical group, where HR even decreased. However, in the Neutral group, the hypothesis was not confirmed; instead, an opposite pattern emerged, with a significant decrease in HR, contrary to our expectations. As for RMSSD, no significant differences were found between the baseline and reaction phases for any of the three natural groups. In contrast, the Neutral group exhibited a significantly higher average RMSSD level at reaction compared to the baseline phase, further supporting our hypothesis.

In summary, although some of these results contradict our initial expectations, they can be interpreted through previous research. For example, Browning et al. [21] found that nature exposure increased physiological arousal, potentially reflecting greater engagement with natural stimuli, which supports positive affect maintenance. This also aligns with Ulrich’s [3] Stress Recovery Theory (SRT), which suggests that exposure to pleasant natural environments can increase, decrease, or have no effect on arousal, depending on the individual’s initial state.

Similarly, Anderson et al. [57] found that physiological arousal levels were comparable between nature and neutral VR conditions, while subjective reports revealed a crucial difference—relaxation in nature versus boredom in the neutral condition. These findings highlight the importance of integrating both implicit and explicit measures to gain a more comprehensive understanding of physiological and psychological responses.

## 5. Conclusions

The aim of this study was to explore the restorative potential of virtual natural scenarios by assessing their psychological and physiological effects in terms of emotional valence and arousal.

The results from self-report measures highlight the ability of virtual natural environments to enhance positive emotions in terms of valence following exposure to arousing stimuli. On the other hand, the analysis of both self-reported and physiological data shows the effectiveness of both neutral and natural scenarios in terms of arousal reduction. Additionally, physiological data reveal that participants in the Natural groups showed higher levels of physiological activation compared to the Neutral group. However, when considering both explicit and implicit measures, it became clear that this arousal was positively perceived in terms of valence, promoting the maintenance of positive affect. This contrasts with the neutral scenario, where lower levels of activation may have been linked to boredom and/or disengagement, ultimately diminishing positive affect in the Neutral group.

From this perspective, the integration of implicit and explicit measures proved to be essential for a comprehensive understanding of the emotional experiences elicited by virtual scenario exposure. These measures have complemented each other, enabling the emotional appraisal attributed to individuals’ physiological activation. Thus, the physiological arousal recorded must be interpreted within the context of participants’ subjective experience, which is an immediate and subjective perception.

### Limitations and Future Directions

In conclusion, the study is constrained by its sample size and the lack of randomization in selecting VR natural environments, while the decision to allow participants to choose their preferred recovery scenario enhanced ecological validity by aligning with individual preferences and contextualized decision-making processes. This user-centered approach [47] to the assessment of emotions and physiological arousal may represent a valuable contribution to the research and interventions aimed at health and wellbeing promotion in multiple contexts, such as workplace, social and therapeutic settings, to prevent the buildup of stress and foster relaxation, or to address stress and anxiety disorders.

Moreover, this approach could be extended to the validation of other types of virtual natural scenarios, incorporated into different types of research and scopes. Moreover, although the perceived levels of a sense of presence and realism within our virtual environments were above average, we recognize the need to further investigate whether a more realistic representation of environments could affect the emotional reactions of participants.

## Data Availability

All the data can be found in the Supplementary.

## Supplementary Materials

The following supporting information can be downloaded at: https://www.mdpi.com/article/10.3390/ijerph22040535/s1, Table S1: Conceptualization of the VR Natural scenarios according to the eight components of the Biophilic Effect [58]; Table S2: Descriptive statistics of IPQ and SUS overall scores; Table S3: Descriptive statistics of IPQ and SUS scores across groups; Table S4: Descriptive statistics of perceived Valence (SAM) component as function of Type of Cycle Factor, Cycle Factor and Group condition; Table S5: Descriptive statistics of Arousal SAM component as function of Type of Cycle Factor, Cycle Factor and Group condition; Table S6: Descriptive statistics of Mean HR as function of Type of Cycle Factor, Cycle Factor and Group condition; Table S7: Descriptive statistics of RMSSD as function of Type of Cycle Factor, Cycle Factor and Group condition. Refs. [58,59,60] is cited in Supplementary Materials.
