# Supplementary material for: Exploring the Restorative Effects of Natural Environments in Virtual Reality
Source: Int J Environ Res Public Health. 2025 Apr 1;22(4):535. doi: 10.3390/ijerph22040535 (PMC12027046; doi:10.3390/ijerph22040535)
Supplement: Supplementary file 1 [file ijerph-22-00535-s001.zip › ijerph-3500390-supplementary.pdf]

Exploring the Restorative Effects of Natural Environments in Virtual Reality

Supplementary Materials

S1. Conceptualization of VR Natural Environments

Table S1. *Conceptualization of the VR Natural scenarios according to the eight components of the Biophilic Effect* [58]

**Biophilic components**	**VR Natural scenarios**		
	Tropical island	***Meadow***	***Forest***
Sunlight	3	3	1
Color	3	2	3
Gravity	3	3	2
Fractal elements	2	2	3
Curves	3	3	3
Details	2	2	3
Water	3	0	2
Life	1	2	3
Total	20/24	17/24	20/24

S2. Preliminary assessment of VR experience

The evaluation of the VR environments' realism and usability was conducted using standardized measures, specifically the Ingroup Presence Questionnaire (IPQ) and the System Usability Scale (SUS). This evaluation was carried out after the entire experience, meaning that no specific data for each VR scenario were considered; instead, the measures reflect participants' general experience with the virtual environments as a whole.

To assess the realism and immersive qualities of the VR environments, the IPQ [59] was employed. This questionnaire measures different aspects of presence within virtual settings, including realism and the sense of being immersed in the environment. Responses range from 0 (completely disagree) to +6 (completely agree). The realism subscale ($\alpha = .741$) specifically captures how lifelike the virtual environments appeared to participants, with an example item stating, "I perceived the scenarios as if they were real". The presence subscale ($\alpha = .836$) evaluates the degree to which participants felt engaged in the virtual setting, as reflected in statements such as "I felt completely involved in the scenarios". Mean scores for the two components of the scale were calculated.

The SUS [60] assesses the perceived usability of a system using a 10-item scale ($\alpha = .658$), with response options ranging from 1 (strongly disagree) to 5 (strongly agree). An example item from the SUS includes statements such as "I found the application very easy to use". The scoring procedure follows the original method by Brooke [60]: for odd-numbered items (1, 3, 5, 7, 9), the assigned score is reduced by 1, while for even-numbered items (2, 4, 6, 8, 10), the score is calculated as 5 minus the participant's rating. The recalculated scores are then summed and multiplied by 2.5, resulting in a final usability score ranging from 0 to 100.

Table S2 provides overall descriptives for the variables of interest, showing that participants rated the VR environments as moderately realistic while experiencing a stronger sense of presence. Despite the moderate realism, the level of immersion suggests that the virtual settings were engaging. Usability was rated highly, indicating that the system was user-friendly and did not present significant technical challenges. Overall, the results suggest that the VR experience was accessible and immersive, justifying its use as an effective tool for the present research.

Table S2. *Descriptive statistics of IPQ and SUS overall scores*

	**Mean**	***SD***
**IPQ Realism**	2.064	1.077
**IPQ Presence**	3.538	1.073
**SUS**	78.510	10.221

To ensure that participants had a similar VR experience across the different groups, a one-way ANOVA was conducted to compare the scores for realism, presence, and usability across the four groups. **Table S3** provides the descriptive statistics for the IPQ and SUS scores across the groups. The results revealed no significant differences between groups for any of the measures, specifically realism, F (3, 26) = 1.97, p = 0.144; presence, F (3, 26) = 1.39, p = 0.267; and usability, F (3, 26) = 0.33, p = 0.803. This suggests that participants across the different groups (Neutral, Forest, Meadow, and Tropical Island) generally had similar experiences in terms of realism, presence, and usability, justifying the comparison of these measures across groups. This supports the idea that the VR experience was consistent, and the effects observed in the study were not due to significant differences in how participants perceived the technical characteristics of the VR scenarios.

Table S3. *Descriptive statistics of IPQ and SUS scores across groups*

	**Group**	**N**	**Mean**	**SD**
**IPQ Realism**	Neutral	14	1.619	0.876
	Forest	12	1.833	0.916
	Meadow	13	2.359	1.182
	Tropical island	13	2.462	1.175
**IPQ Presence**	Neutral	14	3.161	0.880
	Forest	12	3.479	1.475
	Meadow	13	3.692	0.958
	Tropical island	13	3.846	0.916
**SUS**	Neutral	14	78.929	10.411
	Forest	12	80.417	9.098
	Meadow	13	78.077	12.548
	Tropical island	13	76.731	9.264

S3. Descriptive statistics of the variables of interest across cycle, type of war-scenarios and groups

Table S4. *Descriptive statistics of perceived Valence (SAM) component as function of Type of Cycle Factor, Cycle Factor and Group condition*

	NEUTRAL				*FOREST*				*MEADOW*				*TROPICAL ISLAND*			
	Post War scenario		Post Recovery scenario		Post War scenario		Post Recovery scenario		Post War scenario		Post Recovery scenario		Post War scenario		Post Recovery scenario	
	M	*SE*	*M*	*SE*	*M*	*SE*	*M*	*SE*	*M*	*SE*	*M*	*SE*	*M*	*SE*	*M*	*SE*
Party	3.31	.211	3.15	.180	3.67	.220	4.17	.187	3.00	.220	4.42	.187	3.54	.211	4.39	.180
Shelter	2.77	.212	2.85	.242	2.25	.221	3.67	.252	2.33	.221	4.08	.252	2.15	.212	4.15	.242
Medical Camp	2.31	.198	2.77	.249	1.75	.206	4.08	.259	2.00	.206	4.08	.259	1.77	.198	4.07	.249
Wreckage	1.92	.206	2.62	.237	1.42	.214	4.00	.247	1.75	.214	4.25	.247	1.54	.206	4.23	.237
Mass Graves	1.92	.191	2.77	.27	1.50	.199	3.67	.281	1.33	.199	3.83	.281	1.46	.191	3.92	.270
Crossfire	2.39	.208	2.85	.286	1.83	.217	3.67	.297	1.75	.217	4.17	.297	1.92	.208	3.69	.286
Hostages	2.00	.258	2.62	.271	2.00	.268	3.83	.282	1.83	.268	4.08	.282	2.00	.258	3.77	.271
Guerrilla	2.15	.227	2.54	.261	1.67	.236	3.67	.271	1.67	.236	4.00	.271	1.85	.227	3.69	.261
Gunpoint	2.54	.229	2.77	.245	1.83	.239	3.83	.255	2.17	.239	3.83	.255	2.08	.229	4.23	.245

Table S5. *Descriptive statistics of Arousal SAM component as function of Type of Cycle Factor, Cycle Factor and Group condition*

	NEUTRAL				*FOREST*				*MEADOW*				*TROPICAL ISLAND*			
	Post War scenario		Post Recovery scenario		Post War scenario		Post Recovery scenario		Post War scenario		Post Recovery scenario		Post War scenario		Post Recovery scenario	
	M	*SE*	*M*	*SE*	*M*	*SE*	*M*	*SE*	*M*	*SE*	*M*	*SE*	*M*	*SE*	*M*	*SE*
Party	3.154	0.299	2.077	0.211	2.909	0.325	1.818	0.229	2.538	0.299	1.308	0.211	3.154	0.299	1.462	0.211
Shelter	3.154	0.242	2.154	0.255	3	0.263	1.818	0.277	3.231	0.242	1.308	0.255	3.615	0.242	1.692	0.255
Medical Camp	3.231	0.292	2.231	0.244	2.818	0.318	1.909	0.265	2.769	0.292	1.385	0.244	3.692	0.292	1.462	0.244
Wreckage	3.923	0.241	2.077	0.238	3.364	0.262	1.636	0.258	3.462	0.241	1.385	0.238	4	0.241	1.462	0.238
Mass Graves	3.231	0.29	2.154	0.227	2.455	0.315	1.545	0.246	3	0.29	1.462	0.227	3.462	0.29	1.615	0.227
Crossfire	3.308	0.221	2.385	0.203	3.273	0.24	1.364	0.221	3.538	0.221	1.308	0.203	4.077	0.221	1.769	0.203
Hostages	3.308	0.289	2.231	0.194	3.182	0.314	1.455	0.211	3.538	0.289	1.231	0.194	3.231	0.289	1.538	0.194
Guerrilla	3.923	0.231	2.231	0.261	4.091	0.251	1.727	0.283	4.462	0.231	1.538	0.261	4.692	0.231	1.923	0.261
Gunpoint	3.154	0.336	2.308	0.234	3.091	0.366	1.545	0.255	3.308	0.336	1.231	0.234	3.769	0.336	1.385	0.234

Table S6. *Descriptive statistics of Mean HR as function of Type of Cycle Factor, Cycle Factor and Group condition*

	NEUTRAL				*FOREST*				*MEADOW*				*TROPICAL ISLAND*			
	Post War scenario		Post Recovery scenario		Post War scenario		Post Recovery scenario		Post War scenario		Post Recovery scenario		Post War scenario		Post Recovery scenario	
	M	*SE*	*M*	*SE*	*M*	*SE*	*M*	*SE*	*M*	*SE*	*M*	*SE*	*M*	*SE*	*M*	*SE*
Party	89.214	4.077	83.643	3.648	81.909	4.599	81.545	4.116	93.2	4.824	89.4	4.317	93.333	5.085	88	4.55
Shelter	86.286	3.787	81.571	3.643	84	4.272	81.182	4.11	94.5	4.48	92.1	4.31	86	4.723	88	4.544
Medical Camp	84.786	3.417	78.786	3.328	80.909	3.855	78.818	3.755	92.3	4.043	87.9	3.938	88.222	4.262	84.889	4.151
Wreckage	83.214	3.55	79.429	2.882	80.273	4.005	79.182	3.251	92.7	4.2	88.1	3.41	86.889	4.428	84.333	3.594
Mass Graves	83.5	3.482	79.071	2.825	79.273	3.929	77.818	3.187	89.9	4.12	86.9	3.343	85.444	4.343	85.667	3.524
Crossfire	82.286	3.589	80.071	2.862	80.727	4.049	77.818	3.229	91.8	4.247	89.4	3.386	86.222	4.477	84.667	3.57
Hostages	81.571	3.414	78.714	3.109	80.545	3.852	77.273	3.508	91.7	4.04	88.3	3.679	86.222	4.258	83.444	3.878
Guerrilla	82.5	3.347	78.429	2.992	80	3.776	78.091	3.375	93.4	3.96	91	3.54	86	4.175	85	3.731
Gunpoint	82.929	3.608	79.07	3.088	80.727	4.07	79.364	3.483	95.2	4.269	95.6	3.653	87.111	4.5	84.889	3.851

Table S7. *Descriptive statistics of RMSSD as function of Type of Cycle Factor, Cycle Factor and Group condition*

	NEUTRAL				*FOREST*				*MEADOW*				*TROPICAL ISLAND*			
	Post War scenario		Post Recovery scenario		Post War scenario		Post Recovery scenario		Post War scenario		Post Recovery scenario		Post War scenario		Post Recovery scenario	
	M	*SE*	*M*	*SE*	*M*	*SE*	*M*	*SE*	*M*	*SE*	*M*	*SE*	*M*	*SE*	*M*	*SE*
Party	33.014	36.131	74.664	17.151	44.755	40.762	33.3	19.349	121.4	42.751	51.87	20.293	36.689	45.064	44.044	21.391
Shelter	42.921	8.234	50.957	8.61	39.364	9.289	46.273	9.714	32.34	9.743	38.77	10.188	46.3	10.27	48.922	10.739
Medical Camp	39.814	8.206	58.636	23.94	44.4	9.257	43.6	27.008	35.95	9.709	95.28	28.326	39.778	10.235	47.578	29.859
Wreckage	48.436	9.158	83.079	23.191	43.309	10.332	43.927	26.162	38.23	10.836	47.58	27.439	42.7	11.422	45.4	28.924
Mass Graves	69.764	13.798	106.829	30.397	36.509	15.566	43.836	34.293	35.07	16.326	40.01	35.967	41.622	17.209	49.289	37.912
Crossfire	118.957	40.14	76.964	12.427	36.445	45.284	44.455	14.02	37.54	47.495	51.25	14.704	41.256	50.064	44.733	15.499
Hostages	144.15	49.296	118.414	30.836	38.673	55.614	50.791	34.788	41.1	58.328	41.27	36.486	38.233	61.483	44.378	38.46
Guerrilla	100.636	27.289	149.879	53.02	37.127	30.786	53.536	59.814	30.61	32.289	45.63	62.734	46	34.035	46.111	66.127
Gunpoint	106.429	26.966	162.821	49.188	44.364	30.421	36.873	55.492	38.28	31.906	43.95	58.2	38.322	33.632	50.111	61.348

References

58. Salingaros, N.A. Biophilia & healing environments: healthy principles for designing the built world. New York, NY, USA: Terrapin Bright Green. **2015**. Available online: https://www.terrapinbrightgreen.com/wp-content/uploads/2015/10/Biophilia-Healing-Environments-Salingaros-p.pdf (accessed on 15 December 2023).
59. Schubert, T.W. The sense of presence in virtual environments: A three-component scale measuring spatial presence, involvement, and realness. *Z. Für Medien.* **2003**, *15*, 69–71. https://econtent.hogrefe.com/doi/abs/10.1026//1617-6383.15.2.69.
60. Brooke, J. *Usability evaluation in industry*, 1st ed.; Taylor Fr.: Wales, England, 1996; SUS-A quick and dirty usability scale, 4-7. https://doi.org/10.1201/9781498710411-35
